# ED-BioRob: A Neuromorphic Robotic Arm With FPGA-Based Infrastructure for Bio-Inspired Spiking Motor Controllers

**DOI:** 10.3389/fnbot.2020.590163

**Published:** 2020-11-30

**Authors:** Alejandro Linares-Barranco, Fernando Perez-Peña, Angel Jimenez-Fernandez, Elisabetta Chicca

**Affiliations:** ^1^Robotics and Technology of Computers Lab (ETSII-EPS), Universidad de Sevilla, Sevilla, Spain; ^2^Smart Computer Systems Researh and Engineering Lab (SCORE), Research Institute of Computer Engineering (I3US), Universidad de Sevilla, Sevilla, Spain; ^3^Applied Robotics Lab, University of Cadiz, Cadiz, Spain; ^4^Faculty of Technology and Cognitive Interaction Technology Center of Excellence (CITEC) - Bielefeld University, Bielefeld, Germany; ^5^Bio-Inspired Circuits and Systems Lab (BICS), Zernike Institute for Advanced Materials, University of Groningen, Groningen, Netherlands; ^6^Groningen Cognitive Systems and Materials Center (CogniGron), University of Groningen, Groningen, Netherlands

**Keywords:** spike-based motor control, neuromorphic robotics, Dynap-SE, FPGA, SPID, spike-based processing, BioRob, AER

## Abstract

Compared to classic robotics, biological nervous systems respond to stimuli in a fast and efficient way regarding the body motor actions. Decision making, once the sensory information arrives to the brain, is in the order of ms, while the whole process from sensing to movement requires tens of ms. Classic robotic systems usually require complex computational abilities. Key differences between biological systems and robotic machines lie in the way information is coded and transmitted. A neuron is the “basic” element that constitutes biological nervous systems. Neurons communicate in an event-driven way through small currents or ionic pulses (spikes). When neurons are arranged in networks, they allow not only for the processing of sensory information, but also for the actuation over the muscles in the same spiking manner. This paper presents the application of a classic motor control model (proportional-integral-derivative) developed with the biological spike processing principle, including the motor actuation with time enlarged spikes instead of the classic pulse-width-modulation. This closed-loop control model, called spike-based PID controller (sPID), was improved and adapted for a dual FPGA-based system to control the four joints of a bioinspired light robot (BioRob X5), called event-driven BioRob (ED-BioRob). The use of spiking signals allowed the system to achieve a current consumption bellow 1A for the entire 4 DoF working at the same time. Furthermore, the robot joints commands can be received from a population of silicon-neurons running on the Dynap-SE platform. Thus, our proposal aims to bridge the gap between a general purpose processing analog neuromorphic hardware and the spiking actuation of a robotic platform.

## 1. Introduction

Neuromorphic engineering (NE) takes inspiration from biology, more specifically from nervous systems. NE aims to solve engineering problems by mimicking the efficacy and efficiency of central and peripheral nervous systems. The hardware implementation of neuromorphic systems often requires full-custom solutions to solve the desired application. Typical technologies are Application-Specific Integrated Circuits (ASICs) (Chicca et al., 2014), Field Programmable Gate Arrays (FPGAs) (Maguire et al., 2007), or even Field Programmable Analog Arrays (FPAAs) (Rocke et al., 2008). The neuromorphic hardware developed in the last decade can be classified mainly as sensors and neural networks. In the sensors field, the most representative examples are vision sensors (Lichtsteiner et al., 2008; Serrano-Gotarredona and Linares-Barranco, 2013) and their pre-processing (Linares-Barranco et al., 2019), audition sensors (Chan et al., 2007; Jimenez-Fernandez et al., 2017), and olfactory sensors (Koickal et al., 2007). The latest analog neural processors are: Re configurable On Line Learning Spiking (ROLLS) Neuromorphic Processor (Qiao et al., 2015), Neurogrid (Benjamin et al., 2014), High Input Count Analog Neural Network chips (HICANNs) (Schemmel et al., 2008, 2010; Calimera et al., 2013) and Dynamic Neurormorphic Asynchronous Processor [Dynap-SE^1^] (Moradi et al., 2018). Digital implementations include Spiking Neural Network Architecture (SpiNNaker) (Furber et al., 2014), the Loihi digital spiking processors from Intel (Davies et al., 2018), and the TrueNorth chip from IBM (Cassidy et al., 2013).

In order to properly and efficiently integrate spiking neuron arrays and complex neuromorphic architectures, a strategy for communicating spikes was needed in this field. Address-Event-Representation (AER) was proposed as a communication protocol for communicating spikes across neural arrays (Sivilotti, 1991), which is presently a standard in the neuromorphic community. Address-Events (AEs) are digital events with a digital label attached (i.e., the address of the neuron in the array). Timing between events is represented by itself (clockless systems) and the addresses identify the source neuron of the event. The use of mapping tables and switches/routers (Zamarreno-Ramos et al., 2013) allow the routing of events to different destinations, enabling the design of complex and arbitrary neural network topologies.

Today's robotics is not properly adapted or does not offer specific solutions for neuromorphic systems. Available products in the market provide motor controllers such as black boxes, which receive a reference command for targeting a position of a joint or a revolution speed. These controllers communicate with each other through industrial field buses, such as Controller-Area-Network (CAN), which introduces extra latency in the control loop and forces a fixed power consumption (Dominguez-Morales et al., 2011). These systems never provide direct access to the signals that drive the motors of the robots. Typically, these motors are driven by digital circuits using pulse-width-modulation (PWM), which imposes a constant power consumption even when the joint is not moving. Bio-inspired motor control spiking systems (Perez-Peña et al., 2013; Perez-Peña et al., 2017) aim to reduce this power consumption by reducing the signal activity that drives the motors through the application of a Pulse Frequency Modulation (PFM) technique (Jimenez-Fernandez et al., 2012).

In this work, we present a fully neuromorphic robotic arm (from spiking sensors to actuators), whose development started from a version of the BioRob X5 robotic arm (Kirchhoff, 2018) with full-access to both the sensors and actuators, developed as a light arm for industrial/medical safe physical human-robot interaction (pHRI) applications. This robot has 4 degrees of freedom (DoF), driven by 4 direct-current (DC) motors. Each of these motors include an optical encoder sensor that informs about the speed and direction of the motor. An updated and modified version of the spike-based PID controller presented in Jimenez-Fernandez et al. (2012) has been implemented for each joint of this robot. Each joint also includes a position sensor that measures the current state of a robot joint, which is used as ground truth in this work. We focused on the development of a spike-based infrastructure for controlling the robot. To this end, a set of printed circuit boards based on FPGAs and micro-controllers, previously developed by the Robotics and Technology of Computers Lab in Seville, were interfaced to the motors. We allowed the flexibility of communicating the desired position of the joints from a computer (through a USB interface) or from the output of a spiking neural network running in a Dynap-SE platform, in a similar manner to Donati et al. (2018).

To summarize, this paper has the following list of contributions:

- The sPID controller from Jimenez-Fernandez et al. (2012) was designed, configured, and adapted to control the speed of a mobile robot. In this work, the robot uses the same kind of motors (DC motors with optical encoders), although the sPID was adapted and configured to maintain a fixed position of each joint. This is a challenge in the spike-domain.
- Properly configuring the Kp, Kd, and Ki constants for the best behavior of the sPID controllers is another challenge solved in this paper for each joint of this robot, where the mass and movements of a joint affects the dynamic of the others.
- This is the first work, for the best of our knowledge, to represent the motor control of a robotic arm in the spike-domain using PFM. Furthermore, this controller provides an interface for SNN implemented in general hardware, which was demonstrated with the Dynap-SE platform for its justification.

The rest of the paper is structured as follows: section 2 reviews a spike-based proportional-integral-derivative controller that was adapted for this robot, section 3 provides details about the architecture of the robot and its neuromorphic electronics, section 4 describes the experiments and results, and section 5 presents the conclusions.

## 2. Materials and Methods

### 2.1. Spike-Based PID Position Motor Controller

The spike-based PID (sPID) controller is a Proportional-Integrative-Derivative controller completely designed considering the requirements of the spike paradigm. Both, its reference input signal and its output signal to drive the motor are spike-based signals. The PFM modulation is used along the Spike-Signal-Processing (SSP) Building Blocks (Jimenez- Fernandez et al., 2010) to compute the intermediate results and to drive the motors. The reference to the sPID controller designed can be provided by any neuromorphic system with a spiking firing rate as output. The previous work by Jimenez-Fernandez et al. (2012) used the spiking reference to control the speed of the motors which eventually turns into a two independent wheels mobile robot speed-controlled. Therefore, the input frequency of spikes resulted in a fixed speed of the robot. In this manuscript, the controller is updated to control the joint position of the robotic arm.

A classic PID controller has three components that use the error signal to create the control one: the proportional, integrative and derivative terms. In Jimenez-Fernandez et al. (2012), a set of SSP building blocks were developed taking into account the formulation of the Laplace domain (S-domain) as they obey the classic PID formulation (see Equations 1, 2). The basic building blocks are: the Spike-Generator, the Integrate-And-Generate neuron model, the Hold-And-Fire, and the Spike-Expander. The following subsections summarize the key features of the blocks of the controller for a better understanding.

(1) $$\begin{array}{r} x\left(t\right)=K_{p}e\left(t\right)+K_{i}\int e\left(t\right)dt+K_{p}\frac{de\left(t\right)}{dt} \end{array}$$

(2) $$\begin{array}{r} PID\left(S\right)=\frac{X\left(S\right)}{E\left(S\right)}=K_{p}+\frac{K_{i}}{S}+K_{d}S \end{array}$$

#### 2.1.1. Spike-Generator

This block aims to convert a digital value into a PFM signal. The algorithm used is based on the synthetic generation methods proposed in Linares-Barranco et al. (2006a), precisely in a modification of the uniform method (Gomez-Rodriguez et al., 2005). The output spikes are generated by using a counter, driven by the internal clock, and a comparator. The counter has a width of N bits, and spikes are generated whenever the counter output in bit reverse order is below the input. The spike rate can be controlled by the number of bits in the counter and a frequency divider applied to the internal clock. The reverse-order bit-wise operation of this generator produces a regularly spaced distribution of the spikes generated in time. When the counter overflows, the stream of spikes is generated in the same order as before if the input remains the same. It does not produce a uniform distribution of spikes (considering the inter-spike-intervals), but a close one, paying a very low computational price on the FPGA circuit.

#### 2.1.2. Integrate-And-Generate Motor Neuron Model

This block consists of two separate parts: the integrator and the generator. The integrator is implemented with a counter that increases or decreases its value depending on the polarity of incoming spikes. This counter value represents the membrane potential of the modeled motor neuron. The size of the counter (N) affects the membrane potential dynamic range, and it is configurable. The second part of this model is a Spike-Generator, as explained in section 2.1.1. This generator produces a stream of spikes whose firing-rate depends on the membrane potential value. This block produces a fast spiking activity, as observed in biological motoneurons. Equation (3) describes the behavior of this block. The output rate of this block (*out*_*rate*_) depends on the current value of the counter (*count*, which represents the current input spiking rate *in*_*rate*_ accumulation), the size of the counter (*NBITS*), the clock frequency (*f*_*CLK*_), and the clock-frequency divider parameter (*FD*_*GEN*_). The counter can be expressed as an integral (Equation 4). Equation (5) represents the unilateral Laplace transform (S-domain), where the constants are grouped under *K*_*i*_. This transfer function coincides with the integrator part in a PID controller. Therefore, *K*_*i*_ can be expressed as in Equation (6)

(3) $$\begin{array}{r} out_{rate}=\frac{count}{2^{NBITS-1}}\times \frac{f_{CLK}}{FD_{GEN}} \end{array}$$

(4) $$\begin{array}{r} out_{rate}\left(t\right)=\int in_{rate}\left(t\right)\;dt\;\times \frac{f_{CLK}}{2^{NBITS_{i}-1}FD_{GEN_{i}}} \end{array}$$

(5) $$\begin{array}{r} F\left(s\right)=\frac{K_{i}}{S} \end{array}$$

(6) $$\begin{array}{r} K_{i}=\frac{f_{CLK}}{2^{NBITS_{i}-1}FD_{GEN_{i}}} \end{array}$$

#### 2.1.3. Hold-And-Fire

This block merges two spike-based signals into a new spiking signal with a frequency that is the difference of the inputs. When the block is ideal, it waits for an input spike from any of the inputs. This spike is hold until a second spike is received and no output is produced. Once the second spike is received, the block will release the first spike if the polarities are compatible, i.e., both spikes have the same sign. Otherwise, it will cancel both events and no spike will be released.

#### 2.1.4. Spike-Derivative

**Figure 1:**
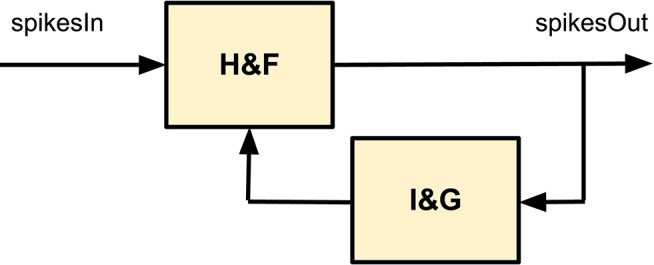
Block diagram of the spike processing block known as “spike-Derivative.” It consists of an Integrate and Generate block connected to a Hold and Fire to close-loop. The result is that the output rate is the derivative of the input rate.

The combination of the Hold-and-Fire and a Integrate-and-Generate blocks can produce a new block whose transfer function performs the derivative component of the PID controller. Figure 1 shows how they are connected to achieve such behavior. Equation (7) shows the resulting transfer function in the S-domain. This equation models the relation between the input/output rate (*F*_*spikesOut*_ and *F*_*spikesIn*_) through the transfer function of the Integrate-and-Generate block [*IG*(*s*)]. In this case, the *K*_*d*_ constant is calculated in a similar way as *K*_*i*_ but taking into account the circuit parameters of this derivative block. Its formulation is shown in (8)

(7) $$\begin{array}{r} \frac{F_{spikesOut}\left(S\right)}{F_{spikesIn}\left(S\right)}=\frac{1}{1+IG\left(S\right)}=\frac{1}{1+K_{d}/S} \end{array}$$

(8) $$\begin{array}{r} K_{d}=\frac{f_{CLK}}{2^{NBITS_{d}-1}FD_{GEN_{d}}} \end{array}$$

#### 2.1.5. Spike-Expander

Previously presented blocks use spike rate as the information carrier. Therefore, the last block of the sPID will represent the motor command. If the time-length of a single spike is extended, the motor command will be different. Thus, this including a spike-expander as the last block will play the role of the proportional part of a PID controller (*K*_*p*_). The output spikes from Integrate and Generate, Hold and Fire and Derivative blocks are narrow in time (1 clock cycle) and they have to be time lengthened in order to allow a motor movement. A DC motor acts as a low pass filter system. Thus, time narrow pulses could not be able to produce the needed force to the motor axis, or they could even be filtered. Therefore, the *K*_*p*_ term of the sPID is related to the amount of time that the spikes are lengthened. Equation (9) shows its calculation, where *SW* is the value of the counter limit configured to extend a pulse for a number of clock cycles. *T*_*CLK*_ is the period of the clock, and *V*_*PS*_ is the voltage of the power supply of the motors (12 v for our ED-BioRob). The higher the *K*_*p*_, the quicker the joint movement.

(9) $$\begin{array}{rc} K_{p} & =\left(SW+1\right)*T_{CLK}*V_{PS} \end{array}$$

#### 2.1.6. Spike-Based PID Position Controller

**Figure 2:**
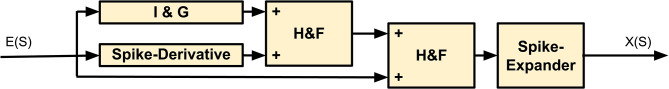
Spike-based PID controller created with SSP building blocks.

The combination of these SSP blocks allows the development of a sPID controller, as shown in Figure 2. The transfer function in the S-domain of the full sPID controller follows Equation (10). In Jimenez-Fernandez et al. (2012), this controller was presented and applied to a mobile platform for speed control. In this paper a modification on the use of the controller is included to properly apply it to several joints of a robotic arm for controlling the angle of each joint and, therefore, a cartesian 3D coordinate of the end effector of the robot in a way closer to biology in the sense of the use of spikes for powering the motors, as a muscle is controlled by a nervous system.

(10) $$\begin{array}{r} PID\left(S\right)=\frac{X\left(S\right)}{E\left(S\right)}=\left(1+\frac{K_{i}}{s}+\frac{s}{s+K_{d}}\right)*K_{p} \end{array}$$

### 2.2. The ED-BioRob Robotic-Arm

The BioRob robot (Lens et al., 2010) is a light robotic arm based on the concepts of elastic and antagonistic actuation, which are inspired by the biological muscle-tendon elastic system. Each joint of the arm has a DC-motor^2^ coupled to ropes and springs as elastic components within the rest of the arm. In this way, each articulation takes properties from progressive and non-linear resorts. Furthermore, the joints are provided with two sensors: a position sensor attached to the DC-motor, which consists of an opto-encoder, and an angular sensor to measure the absolute angle of the joint. More details of the design of this robot can be found in Moehl (2000) and Klug et al. (2008).

**Figure 3:**
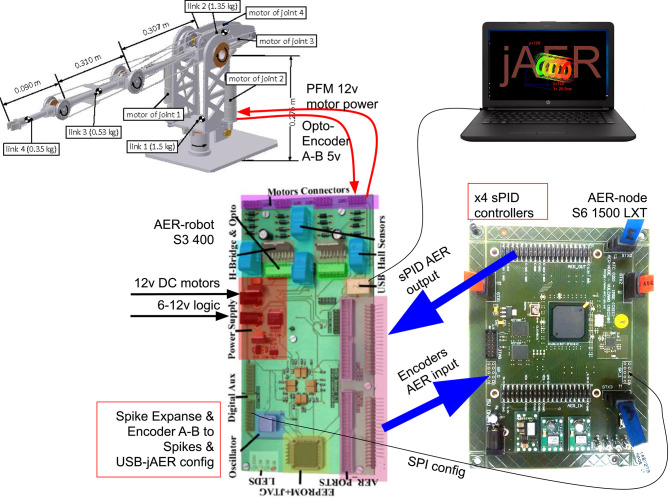
BioRob structure.

Figure 3 shows the actual position of each DC-motor in the architecture of the arm. The position sensors and the encoders attached to the motors ensure cartesian resolution below 1 mm. The position sensors of the joints can measure the elastic forces by means of characteristic curves of rigid joints. This property enables the force control, collision detection, and the proper reaction to them.

**Figure 4:**
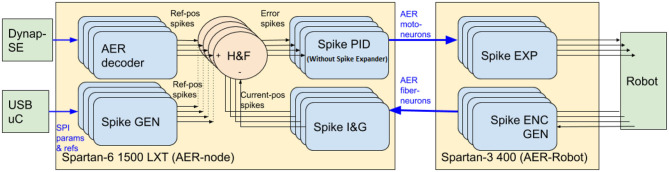
Spike-based PID controller for BioRob split in two FPGA platforms. The sPID without the Spikes Expander is implemented on the Spartan 6 and the Spikes Expander block is implemented on the Spartan 3 platform.

In order to apply a position control technique to the joints of the BioRob arm with the sPID controller, Jimenez-Fernandez et al. (2012) a new Integrate-and-Generate block was included at the output of the optical encoder sensor of each motor. This block makes compatible the output from the encoders with the spiking reference signal. Thus, the error signal *E*(*S*), which is used as the input for the sPID, can be computed. Figure 4 shows a block diagram of the architecture implemented for the ED-BioRob. The SSP building blocks are implemented using two different platforms due to the limited hardware resources of the FPGAs included on the boards. The AER-Robot (Linares-Barranco et al., 2006b), with a Spartan-3 FPGA, is used to interface the arm: (1) the DC motors are driven using PFM signals and (2) the feedback from the opto-encoders, which are conveniently converted into a spiking signal; and (3) the position sensors are received and used as ground truth. This board includes the last SSP block of the sPID, i.e., the Spike-Expander. It also has a state machine in charge of reading the sensors and doing its spiking conversion into AER for the sPID feed-back.

The second platform is the AER-Node (Yousefzadeh et al., 2017), which includes a Field Programmable Gate Array (FPGA) with more resources: a Spartan-6. This board includes the needed sPIDs (without the Spike-Expander block), the Integrate-and-Generate blocks to obtain the current position of each joint, the Hold-and-Fire block to obtain the error signals and a Spike-generator to eventually convert a reference digital input value to spike frequency signal used as reference position of sPIDs, which are received from the software platform jAER^3^. Moreover, a decoder allows controlling the robot joints from an external neuromorphic processor, such as Dynap-SE in this work. This decoder splits the neuromorphic processor output into a set of four spiking reference signals, in our case, for controlling ED-BioRob joints. AER buses were used to connect both boards^4^.

Once the sPID controllers are deployed in this infrastructure of boards for all the robot's joints with the close-loop modified by the inclusion of another integrator for the joints' position control, the proper configuration of *K*_*p*_, *K*_*i*_, and *K*_*d*_ must be done before explaining the experiments. In order to adjust these parameters, in this work, we configured them to obtain the same speed and precision per joint. Table 1 shows the configured parameters.

**Table 1:** *K*_*p*_, *K*_*i*_, *K*_*d*_ parameters of the 4 sPID controllers of the ED-Scorbot joints and the close-loop integrator (*CL*_*i*_).

**Joints**	**SW**	***K*_*p*_**	***NB*_*i*_**	***FD*_*GEN*_*i*__**	***K*_*i*_**	***NB*_*d*_**	***FD*_*GEN*_*d*__**	***K*_*d*_**	***NB*_*CL*_*I*__**	***FD*_*GEN*_*CL*_*i*___**
J1	1,024	2.5e-4	18	4,096	9.3e-2	22	4,096	5.8e-3	18	16
J2	1,024	2.5e-4	18	4,096	9.3e-2	22	4,096	5.8e-3	18	16
J3	512	1.2e-4	18	4,096	9.3e-2	22	4,096	5.8e-3	18	16
J4	512	1.2e-4	18	2,048	0.186	22	2,048	1.2e-2	18	16

*NB is bit length, FD is frequency divider value and SW is spike width*.

## 3. Experiments

### 3.1. Robot Characterization

A set of experiments were performed to characterize the robot. Firstly, a set of references were given to each joint sPID controller of the robot to check the range of the sensors, for each joint limits. The reference given to the joint is a spike rate produced by a digital module included into the FPGA. Equation (11) shows the relation between the input (16-bit integer value) and the spike rate produced by the spike-generator module that generates the input signal for the spike-based control system (named as Ref-pos spikes on Figure 4).

(11) $$\begin{array}{r} rate=\frac{f_{CLK}}{2^{n-1}}Input \end{array}$$

**Figure 5:**
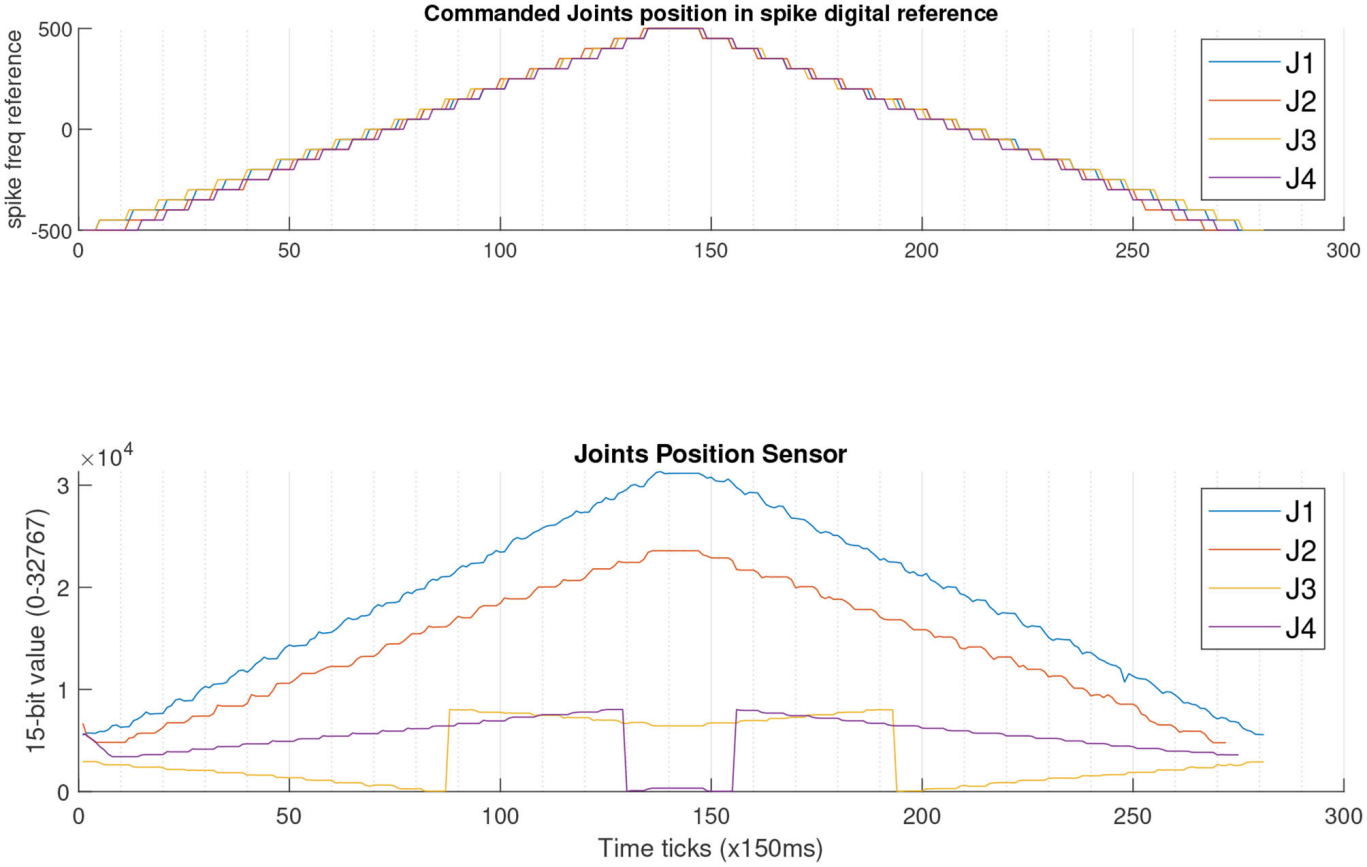
Characterization of joints 1–4. For each joint: the first row shows the commanded position to the joint and the second row shows the raw data read from the sensor.

Figure 5 shows the behavior of each joint when applying a reference signal only to the selected joint. The home position of the robot was considered along the work as having the arm completely vertical with an angle of 90° with respect to the floor. A negative polarity spike reference signal will move the joint from 90 to 0° and a positive polarity will move the joint from 90 to 180°. Therefore, if the home position is considered as 0°, the entire movement would be from −90 to 90°. The stimulus is shown in the third row of each subfigure. This stimulus was generated using Equation (11) with an *Input* ranging from −500 to 500, a 16-bits register (*n* = 16) and a clock frequency of 50 MHz. Therefore, the *rate* represents a spike-based signal with negative polarity from 762.94 Kspikes/s to 0 spikes/s and positive polarity from 0 spikes/s to 762.94 Kspikes/s. The robot was at the home position when this test started, which explains the left part of each graph where the joint movement from home to the −500 digital spike reference can be seen. This range is scanned with 20 steps (a step of 50 digital reference), which represents a spike frequency step of 76.3 Kspikes/s given to the joint every 1 s, as can be seen at the top of the figure. Table 2 summarizes the robot joint's angles and spike reference signals. Joints 3 and 4 sensors are not properly aligned to the robot according to the selected home position, which explains the overflows/underflows in their graphs.

**Table 2:** Values for the experiments to characterize the joints (Ji) and their sensors (Si).

**Dig. Ref**.	**J1 (degrees)**	**S1 (degrees)**	**J2 (degrees)**	**S2 (degrees)**	**J3 (degrees)**	**S3 (degrees)**	**J4 (degrees)**	**S4 (degrees)**
−500	−88.93	−108.63	−86.1	−83.87	−106.52	42.02	−122.12	−43.98
−400	−71.38	−116.21	−76.3	−83.52	−85.38	40.3	−104.18	−44.58
−300	−56.37	−86.80	−57.9	−63.53	−60.18	31.15	−78.45	−33.23
−200	−29.21	−58.46	−39.1	−41.9	−35.63	21.52	−50.93	−21.89
−100	−18.95	−29.75	−19.9	−22.75	−17.08	11.44	−20	−10.92
0	0	0	−1	0	1.69	0	0	0
100	18.82	28.21	15.2	20.11	20.91	−11.37	14.38	10.86
200	34.46	56.73	34	42.02	36.56	160.37	33.27	21.96
300	52.8	85.04	53.2	63.73	55.82	151.06	63.29	34.24
400	70.31	114.74	71.8	82.91	77.38	141.63	86.46	45.41
500	91.06	137.90	88.6	102.58	96.64	130.41	109.64	−124.72

*The results of the movements are shown in Figure 5. The values of both the joints and the sensors are expressed in degrees(°). The values of the angles are measured using video tools. All the sensors values are referenced to the home position (0°). The differences between the angles measured with the joint sensor and the video tool can be understood due to the inertia of the iterative test performed*.

Secondly, an experiment was performed to test the accuracy of the controller when commanding the joints to a particular angle. For this experiment, we selected joint 2, which is the one supporting most of the weight of the arm.

**Figure 6:**
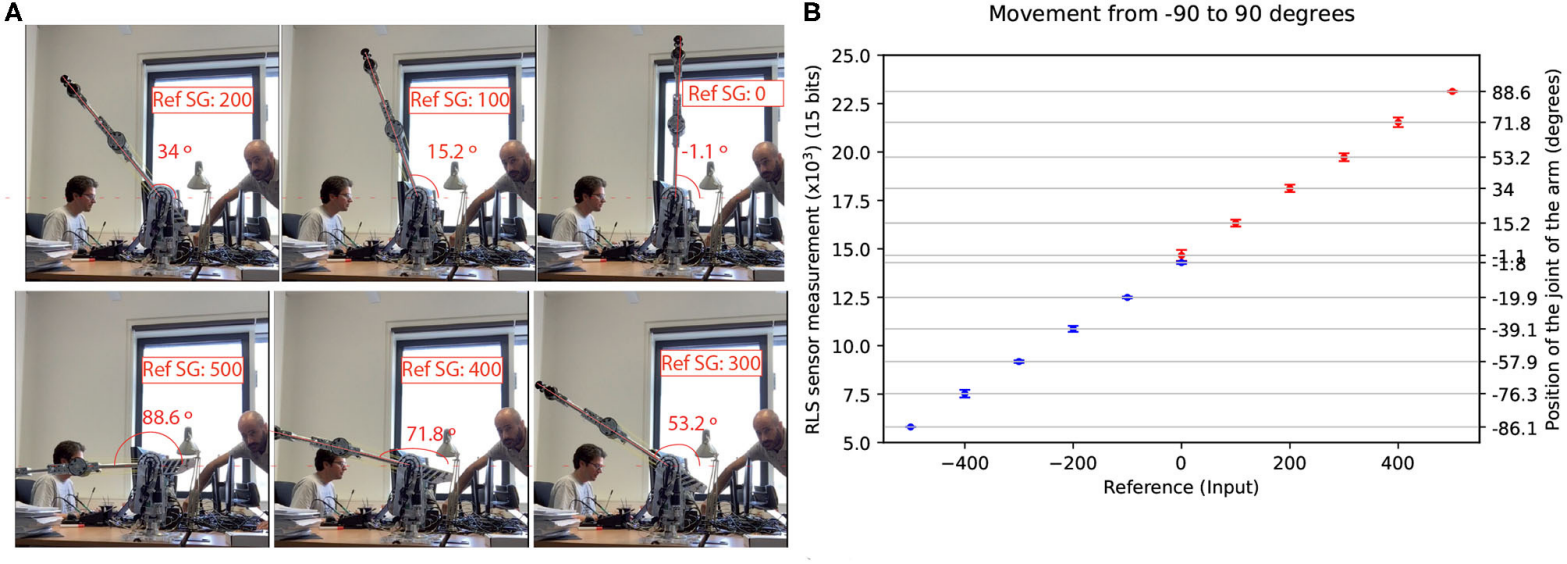
**(A)** Both the input reference to the FPGA (labeled as Ref. SG) and the angle, are shown. The home position of joint 2 is vertical 0°. **(B)** The experiment was run four times for each position. The step was 18°, and for each of them, the mean and standard deviation are shown. Two colors are used to distinguish between positive and negative reference and to show the difference in the mid position (0°). The error bars represent the RMSE. **(A)** Measurement of the angles reached by joint-2. **(B)** Positions reached by the joint when moving from −90 to 90°.

A set of six reference values where given to the controller to move the joint within the range from 0 to 90° repetitively as the first part of the test. Then, the same experiment was conducted for the range −90 to 0° without resetting the controller or re-configuring the home position. These references represent an 18° step. Figure 6A shows how the ground-truth angles were measured: the movement of the joint was recorded and the angles were measured using offline software. This was done in order to validate attached position sensors offsets and quality. Figure 6B shows the performance of adapted spike-based PID controller for the joint 2 position control. The RMSE of the first range (0–90°) is 1.61° (red marks in the figure) and the RMSE of the second range (−90 to 0°) is 3.3° (blue marks in the figure). The standard deviation is higher for the center point (0°) and for both ends of the experiment (90 and −90°). For the sake of clarity, the reference in Figure 6B is represented as the digital input provided to the FPGA.

### 3.2. Trajectory Planning

**Figure 7:**
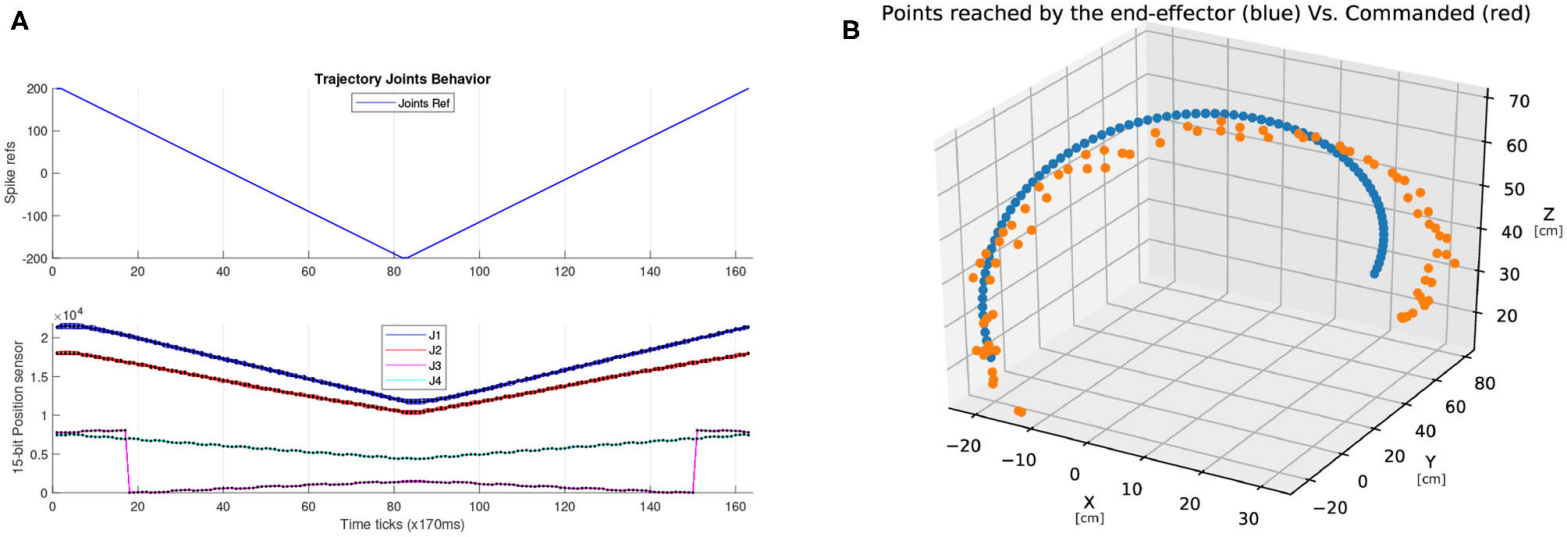
**(A)** Ten iterations of a scan movement for all joints in parallel from −200 to 200 spike refs. The units of both X axis are in ticks of 170 ms. The behavior of both J1 and J2 (blue and red traces of the upper plot) shows that the difference between them is increasing for each repetition of the test. **(B)** 3D representation of the end-effector trajectory (in average for all the runs) for the experiment of **(A)**. Units are in cm. There is a video included as Supplementary Material showing the entire 3D representation of all runs.

Figure 7A shows the behavior of the robot performing a trajectory by moving all the joints at the same time when their stimuli go from a digital reference of −200 to 200 with unitary steps (up to 152.6 Kspikes/s in both polarities). This experiment shows that there is an incremental error of 2.39% considering the measurements of the position sensors of joints 1 and 2 (blue and orange traces) over 10 iterations. The behavior of joint 4, in this shorter range, also includes the overflow of the 15-bit internal counter of the sensor mentioned in Figure 5 experiment due to the offset of the sensor alignment.

Due to the bit truncation errors and considering the physical restrictions due to the surroundings, Figure 7B shows the performance of the arm when a trajectory is given to it. The blue trace shows the trajectory commanded and the orange dots represent the position of the end-effector of the robot during the 10 iterations of the experiment. The average error is 6.6°. All the details for these values are given in Table 3.

**Table 3:** References given to the joints in the experiment shown in Figure 7B.

**Dig. Ref**.	**Ref.(ksps)**	**Joint 1(degrees)**	**Joint 2(degrees)**	**Joint 3(degrees)**	**Joint 4(degrees)**	**X(cm)**	**Y (cm)**	**Z (cm)**
−200	−305.17	−49.43	−38.14	−46.45	−76.71	−14.09	−25.10	12.06
−150	−228.88	−37.42	−31.12	−36.25	−64.93	−19.53	−23.53	25.53
−100	−152.59	−23.40	−18.96	−26.93	−42.60	−20.19	−13.63	46.65
−50	−76.29	−9.33	−9.02	−11.11	−30.47	−10.56	5.16	64.28
0	0	4.62	2.03	−0.38	−6.73	5.70	28.78	70.40
50	76.29	18.70	12.77	10.60	11.11	21.10	51.79	62.34
100	152.59	35.96	23.69	21.72	22.93	31.24	70.38	43.05
150	228.88	49.42	33.83	32.40	46.23	26.24	81.38	22.47
200	305.17	55.30	37.84	33.90	59.81	23.01	82.61	15.93

*References are in Kspikes/s, angles are in degrees(°) and cartesian coordinates in cm. The Dig. Ref. is the value that should be given to the Spikes Generator block that will generate the rate given by the field Ref*.

**Figure 8:**
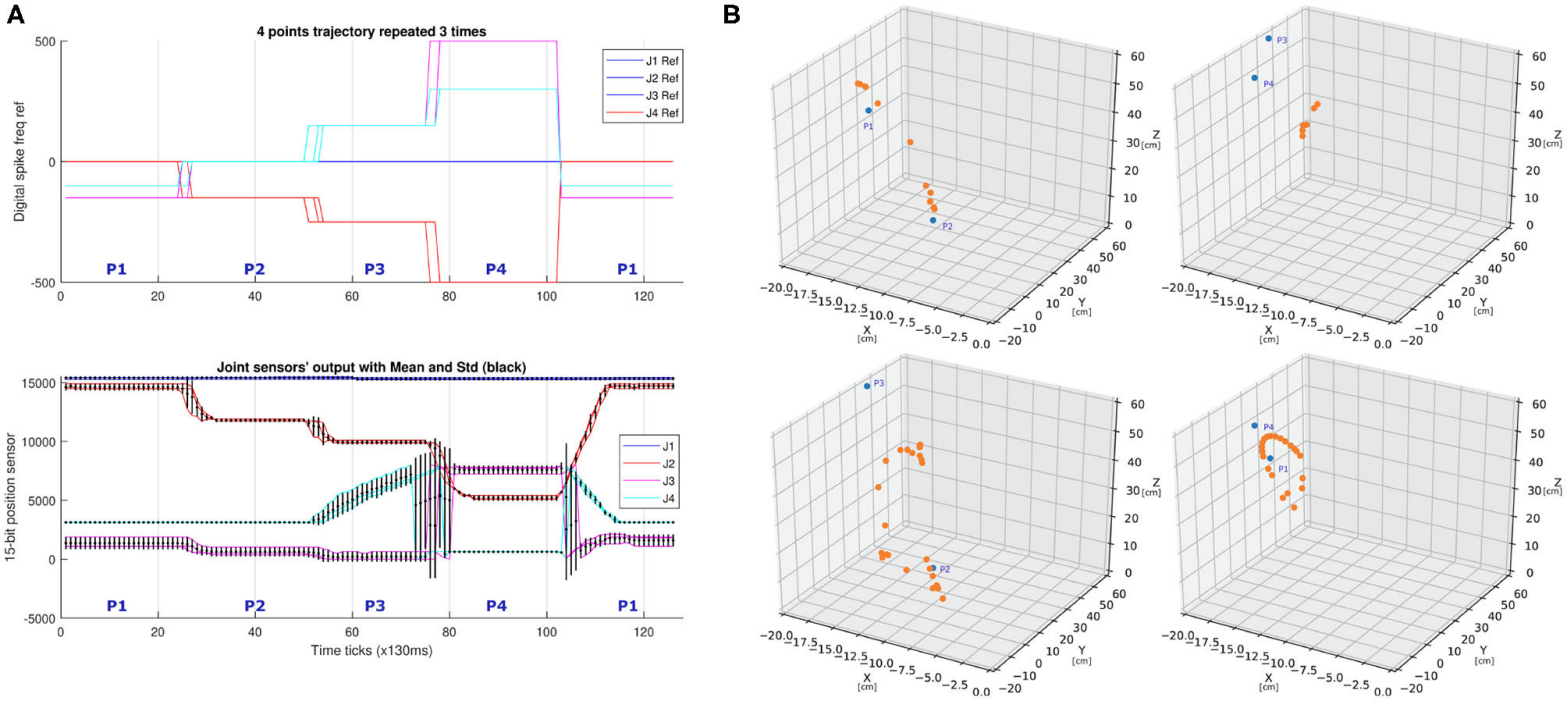
**(A)** Three repetitions of a sequence of four points in space (joint 1 not used) superposed. Units are ticks of 130 ms. **(B)** 3D representation of the trajectory segments followed by the robotic arm among four points. The four plots represent in blue the transitions of the arm between the commanded positons (P1 → P2, P2 → P3, P3 → P4, and P4 → P1 segments). Reached positions are in orange. Commands are sent at 0.25 Hz and measurements are done at 6.5 Hz. All the axes are in cm.

Another trajectory experiment is shown in Figure 8, where the behavior of the arm moving in a 2-D plane is represented. The joint located at the base of the arm was not used in this experiment. The stimuli to each joint are shown in the third row of the figure and the trajectory followed by the arm is shown in Table 4. In this experiment, the trajectory is commanded point by point in a cyclic way with a pause of 1.5 s between points. Since this robot has elastic joints and the sPID controller constants (Kp, Ki, Kd) were adjusted manually to have a quick response, it caused small oscillations around the commanded position for periods of time that were longer than 1.5 s for each 2D position change of the end-effector. Due to this effect, in Figure 8B, the commanded points (blue) and the points reached by the robot (orange) did not coincide during the robotic arm movement between two consecutive points (initial and final).

**Table 4:** Average values given to the joints in the experiment shown in Figure 8.

**Ref. J2**	**J2**	**S2**	**Ref. J3**	**J3**	**S3**	**Ref. J4**	**J4**	**S4**	**(x, y, z)**
0	−1	5667.69	−150	−36.25	1363.82	−100	−20	3470.47	(−12.01, 13.42, 49.65)
−150	−31.12	10154.28	0	1.69	558.34	0	−6.73	3129.42	(−11.26, −1.19, 46.55)
−250	−48.5	12023.23	150	32.4	39.12	150	46.23	5320.04	(−13.38, −4.37, 55.33)
−500	−86.1	14502.67	500	96.64	7808.23	300	63.29	631.61	(−4.55, −20.30, 18.84)

*References are the digital ones, the values of the sensors are shown as they are read, the angles of each joint (J_i_) in degrees(°) and the cartesian coordinates in cm*.

### 3.3. Dynap-SE Control of the Robot

In this experiment, we connected the spiking neuromorphic processor Dynap-SE platform to the robot controller to command a position for a joint using the neuron populations from the four chips of the system. Each Dynap-SE comprises four chips, each having four cores with configurable connectivity of neural populations. In order to extract the population activity from the Dynap-SE through its AER port, its internal FPGA circuit code was modified. In the robot controller, we inserted an AER-Decoder that connects the Dynap-SE output to the input reference for the sPID controller. The use of this decoder can be selected through the jAER interface. In this experiment, we show results for joint 4. We tested different neuron activities from each chip in order to produce the combined activity required to produce a reference PFM signal in the controller to change the angle of the joint. The sPID controller parameters can be modified to reduce and adjust the population activity needed to move the angles if the application requires it.

**Figure 9:**
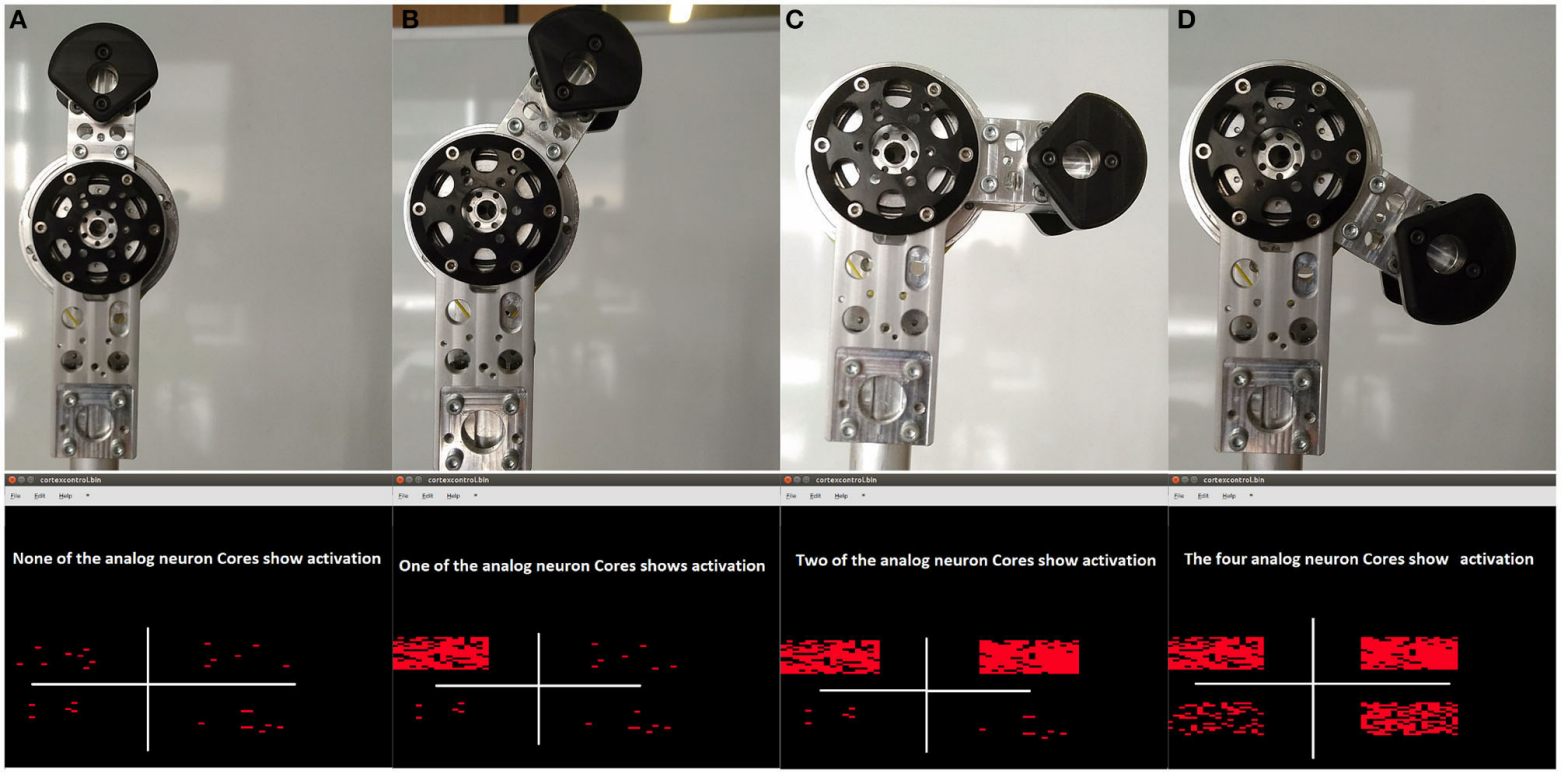
(Top): From left to right, robot joint positions from 0^*o*^ to 130^*o*^. (Bottom): Dynap-SE software interface with neurons activity for commanding from 0^*o*^ to 130^*o*^. The figures of the second row show only a section of the software used to interface Dynap-SE. The plots are included only to highlight how the activity (red dots) of the populations implemented on Dynap-SE changes within the range of the joint. **(A)** Home, 0^*o*^, **(B)** 30^*o*^, **(C)** 90^*o*^, **(D)** 90^*o*^.

Figure 9 shows the results with four different angles of joint 4 and the Dynap-SE software interface that produces the required PFM reference signals for the FPGA embedded sPID controller by joining several neuron-populations activity. Each Core 0 of the four chips was used for joint 4, while the other three cores of all chips were available for the other joints of the robot.

## 4. Conclusions

A spike-based proportional-integral-derivative speed motor controller was adapted to control the position of the 4 joints of a light and safe physical human-robot interaction (pHRI) robotic arm, called ED-BioRob. These sPID controllers were deployed in two FPGA platforms, i.e., the AER-Robot and the AER-Node boards, which provide Address-Event-Representation interfaces for spiking systems and can drive DC motors with Pulse Frequency Modulation signals, mimicking the motor-neurons of mammals. The system allows receiving the reference signals for the joints from a computer, through USB and the open-source software jAER; or from a neuromorphic processor (DYNAP-SE) executing a spiking neural network. The experiments conducted in this work show that the sPID offers the worst RMSE of 3.3° after several iterations of joint movements from −90 to 90°. The system is totally functional for performing point-by-point trajectories. It was demonstrated that the robot can be commanded through a population of silicon neurons. Future works will aim to use the robot in learning-based applications on the spike-domain to implement new neuro-inspired motor controllers for human-robot natural interaction. The robot and its FPGA-based infrastructure will serve as a testing platform for neuromorphic engineers.

## Data Availability Statement

The original contributions presented in the study are included in the article/Supplementary Material, further inquiries can be directed to the corresponding author/s.

## Ethics Statement

Written informed consent was obtained from the individuals for the publication of any potentially identifiable images included in this article.

## Author Contributions

AL-B and AJ-F developed the hardware. AL-B and FP-P performed the experiments and wrote the paper. EC supervised the work related to the DynapSe and the BioRob. AJ-F and EC checked the paper. AL-B and EC provided the funds. All authors contributed to the article and approved the submitted version.

## Conflict of Interest

The authors declare that the research was conducted in the absence of any commercial or financial relationships that could be construed as a potential conflict of interest.

## Footnotes

^1^https://www.ini.uzh.ch/en/research/groups/ncs/chips/Dynap-se.html (accessed July 31, 2020).

^2^Graphite Brushes DC motors from Maxom with planetary gearhead and attached MR encoder.

^3^http://jaerproject.org

^4^Source files are available on a Git repo at: https://github.com/RTC-research-group/EDIP_library/tree/master/ED-BioRob (accessed September 15, 2020).
